# Presence of *TERT* Promoter Mutations is a Secondary Event and Associates with Elongated Telomere Length in Myxoid Liposarcomas

**DOI:** 10.3390/ijms19020608

**Published:** 2018-02-18

**Authors:** Monica S. Ventura Ferreira, Martina Crysandt, Till Braunschweig, Edgar Jost, Barbara Voss, Anne-Sophie Bouillon, Ruth Knuechel, Tim H. Brümmendorf, Fabian Beier

**Affiliations:** 1 Department of Hematology, Oncology, Haemostaseology and Stem Cell Transplantation, RWTH Aachen University Medical Faculty, 52074 Aachen, Germany; mventuraferreira@ukaachen.de (M.S.V.F.); mcrysandt@ukaachen.de (M.C.); ejost@ukaachen.de (E.J.); bavoss@ukaachen.de (B.V.); abouillon@ukaachen.de (A.-S.B.); tbruemmendorf@ukaachen.de (T.H.B.); 2 Institute of Pathology, RWTH Aachen University Medical Faculty, 52074 Aachen, Germany; tbraunschweig@ukaachen.de (T.B.); rknuechel-clarke@ukaachen.de (R.K.)

**Keywords:** myxoid liposarcoma, sarcoma, telomere, telomerase, *TERT* promoter mutation

## Abstract

The occurrence of *TERT* promoter mutations has been well described in soft tissue sarcomas (STS). However, the biological role of these mutations as well as their impact on telomere length in STS is still unclear. We analyzed 116 patient samples diagnosed with 22 distinct histological subtypes of bone and STS for the occurrence of *TERT* promoter mutations by Sanger sequencing. We observed *TERT* promoter mutations at an overall frequency of 9.5% distributed over 7 different sarcoma subtypes. Except for one chondrosarcoma case harboring a C250T mutation, all other mutations were detected at location C228T. By far the far highest frequency of *TERT* promoter mutations was found in myxoid liposarcoma (MLS) (4 out of 9 cases studied, i.e., 44%). Assessment of telomere length from tumor biopsies revealed that *TERT* promoter-mutated MLSs had significantly fewer shortened telomeres in comparison to *TERT* wildtype MLSs. Based on the frequency of *TERT* promoter mutations and the elongated telomere length in mutated compared to wildtype MLS, we hypothesize that occurrence of *TERT* promoter mutations has a pivotal role in the disease progression as a secondary genetic event at a time when tumor cells face the need for telomere elongation to allow further proliferation.

## 1. Introduction

Telomeres are repetitive DNA sequences protecting the ends of chromosomes. Telomeres shorten with each cell division, and telomere length (TL) limits the replicative capacity of a cell once telomeres become critically short [1,2]. Telomere shortening can be counteracted by the expression of telomerase (*TERT*), an enzyme that can (re-)elongate telomeres [3,4] a phenomenon that can also be used therapeutically in vivo [5]. One of the hallmarks of cancer is unlimited cell division [6]. Previous studies showed that tumor cells mostly maintain their telomere length by increased telomerase expression [7] and rarely by a mechanism called alternative lengthening of telomere (ALT) [8]. However, the exact mechanism how tumor cells increase telomerase activity is incompletely understood and varies between the different tumor entities [9].

Recently, two highly recurrent mutations in the promoter region of *TERT* (c.-124 C>T and c.-146 C>T), also called C228T and C250T, respectively were described in 71% of all melanomas [10,11]. In follow up studies, these mutations were reported to be, overall, the most prevalent somatic point mutations being present at varying frequencies in over 50 different types of cancers [12,13,14,15,16]. Of note, both the C228T and the C250T mutation are typically heterozygous, mutually exclusive, and both create the identical 11bp sequence (i.e., “CCCGGAAGGGG”) that has substantial similarity to an ETS binding motif representing a de-novo binding site for an activating ETS family transcription factor. Both mutations activate *TERT* promoter activity and *TERT* gene transcription functionally resulting in in vitro telomere elongation [10,11].

Soft tissue sarcomas (STS) represent a heterogeneous disease with more than 50 clinically relevant subtypes with different histologies, molecular-genetic profiles, tumor-location patterns, and prognoses [17,18,19]. Genomic alterations including chromosomal translocations, DNA copy number variations and the presence of typical genetic aberrations are hallmarks of certain subtypes of sarcomas [20]. One of the molecularly best-studied subtypes is myxoid liposarcoma (MLS). In 95% of the cases, MLSs share the reciprocal translocation t(12;16)(q13; p11). The breakpoint on chromosome 12 involves the transcription factor CHOP (C/EBP homologous protein), member of the CAAT/enhancer-binding protein (C/EBP) family, a gene involved in adipocyte differentiation [21,22]. The CHOP protein presents a leucine zipper dimerization domain that can bind C/EBP proteins and block the interaction with DNA [21,22]. The breakpoint on chromosome 16 involves the FUS/TLS (Fused in Sarcoma/Translocated in Sarcoma) gene, an RNA-binding protein and member of the TET protein family that also includes the EWS protein. The main characteristic of this chromosomal translocation is that it results in the expression of the fusion transcript TLS/FUS-CHOP. Functionally, the resulting FUS-DDIT3 fusion protein induces increased expression of the C/EBP promoting oncogenic activation and tumor formation [23,24,25]. Based on the oncogenic potential and respective mouse models [26], the occurrence of t(12;16)(q13; p11) translocation is generally expected to be the first event in sarcoma initiation in MLS.

In the literature, only a few studies exist on telomeres or *TERT* promoter mutation status in sarcoma. These include the introduction of telomere length as a prognosis marker in Ewig sarcoma [27], the association of telomere length of peripheral blood leukocytes with increased risk for soft tissue sarcoma [28], telomere length in complex and simple karyotype sarcomas [29], assessment of ALT in sarcoma [30,31,32,33,34], mosaicism of telomere maintenance mechanisms in sarcomas [35,36] and the frequency of *TERT* promoter mutations in sarcomas [13,37,38,39]. However, no study has so far correlated *TERT* promoter status and telomere length in bone and STSs.

The aim of our study was first to investigate the prevalence of *TERT* promoter mutations in the different sarcoma subtypes and second to analyze the relation between presence of *TERT* promoter mutations and telomere lengths in a specific subtype of sarcomas, the MLSs.

## 2. Results

### 2.1. TERT Promoter Mutations in Bone and Soft Tissue Sarcomas (STS)

**Figure 1:**
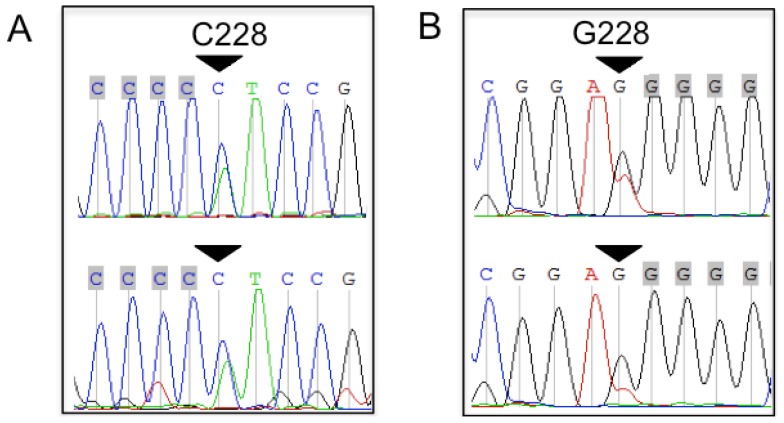
Representative chromatograms of heterozygous *TERT* promoter mutations in two bone and soft tissue sarcomas. Sequencing direction: (**A**) sense, (**B**) antisense. An arrow indicates mutations.

First, we analyzed for the incidence of *TERT* promoter mutations in 116 bone and STS tumors of 22 different subtypes (Table 1). We found mutations in 11/116 patients (9.5%), namely in four cases of MLS, one solitary fibrous tumor (SFT), two malignant fibrous histiocytomas (MFH) or undifferentiated pleomorphic sarcoma (UPS), one pleomorphic sarcoma, one poorly differentiated sarcoma NOS and two chondrosarcomas (Figure 1; Table 2). Of note, 10 out of 11 (91%) mutations found were C228T and only in one patient with chondrosarcoma we observed a *TERT* promoter mutation at position C250T (Table 3). In Table 1, mutation frequencies are summarized in a side-by-side comparison with different studies reported in the literature.

### 2.2. Tumor Grading Correlates with the Presence of TERT Promoter Mutations

We next focused on whether the presence of *TERT* promoter mutations correlated with clinical features of our sarcoma cohorts. We did not observe any significant differences comparing the initial tumor localization: head/neck 10% (1/10), extremities and superficial trunk 18% (5/27), retroperitoneal 0% (0/18), abdomino-thoracic 4% (1/24) or others 13% (4/30). Since the histological grading is one of the most important factors for clinical decision-making, we compared patients according to histological grading. Patients with histopathological G1 grading/well-differentiated tumors showed significant higher percentage of *TERT* promoter mutations (5/21, 24%) compared to patients with G2-3 grading/poor differentiation (6/92, 7%, *p* = 0.030, see Table 2). No further clinical characteristics such as age, gender, race, disease progression status or development of metastasis (Table 2) differed significantly among WT and *TERT* promoter-mutated patients.

### 2.3. Myxoid Liposarcomas (MLSs) Exhibit Longer Telomeres than Wildtype Tumors

Next, we focused on MLS based on the incidence of *TERT* promoter mutations—ranging in the literature from 22% to 79% (Table 1)—as well as their homogenous genetic background characterized by the translocation t(12;16)(q13; p11), a genetic hallmark of MLS. Among the wildtype tumors, MLS only one (1/5 = 20%) presented distant metastasis in contrast to 2 out of 2 (100%) *TERT* promoter-mutated tumors from which patient clinical data was available.

**Figure 2:**
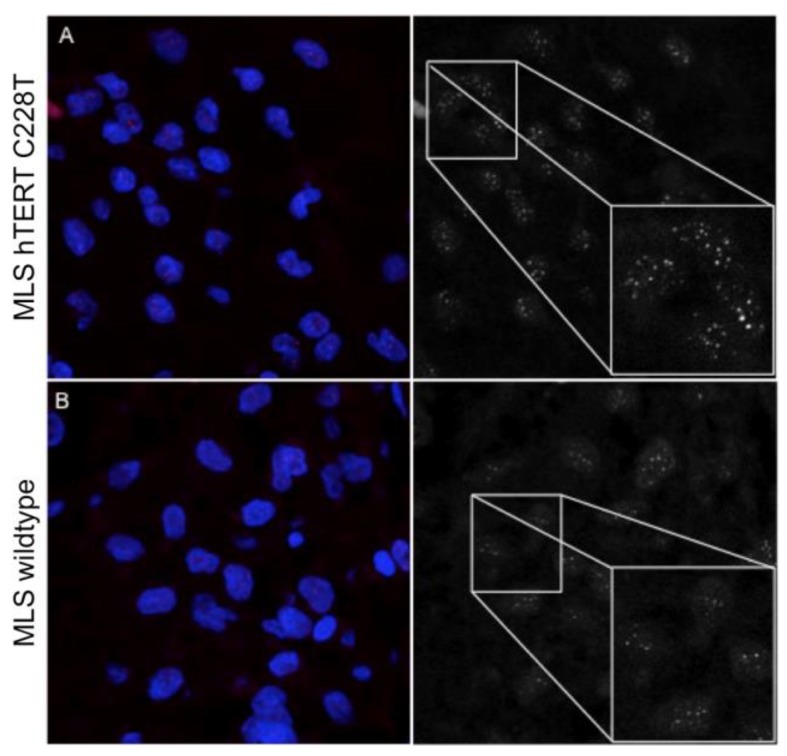

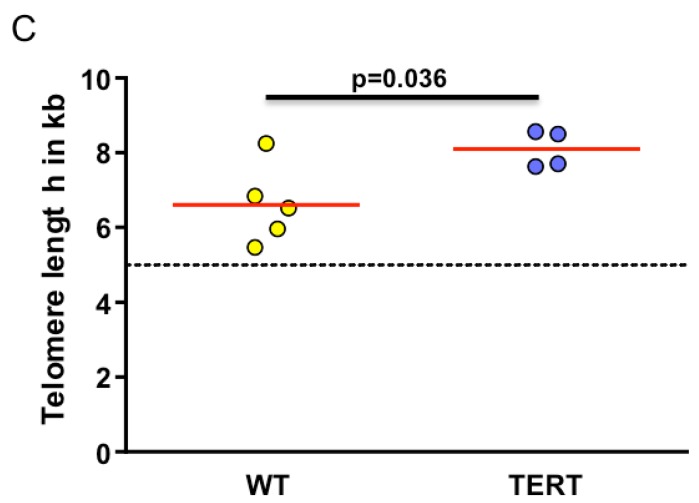
Representative Q-FISH images captured by confocal laser scanning microscopy: (**A**) C228T *TERT* promoter-mutated myxoid liposarcoma, and (**B**) a *TERT* promoter wildtype myxoid liposarcoma patient. Magnification 630×. (**C**) Quantification of telomere length after Q-FISH analysis (arbitrary units, a.u.). The dashed line represents 5 kb, the threshold of critical short telomeres.

Since telomere length declines with age, the comparison of the age of the patients did not reveal significant differences between MLS wildtype (61.4 ± 6.0 years, *n* = 5) and *TERT* promoter-mutated tumors (49.5 ± 10.6 years, *n* = 4; *p* = 0.33). Interestingly, telomere length of MLS samples with *TERT* promoter mutations was found to be significantly less shortened (8.10 ± 0.50 kb; *n* = 4) compared to the telomere length of wildtype tumors (6.61 ± 1.06 kb; *n* = 5; *p* < 0.037) (Figure 2A–C). No statistically significant correlations between elongated telomere length and age, gender, tumor location, tumor grade, disease progression, and development of metastasis were observed likely due to small sample size. Assessment of ALT revealed no evidence of significant heterogeneity of telomere sizes, which would reveal the presence of ALT, in either *TERT* promoter MLS or wiltype tumors (Figure S1).

## 3. Discussion

Our study introduces the third largest cohort of bone and STS analyzed for *TERT* promoter mutations among literature [37,38] and the first systematic analysis of telomere length in *TERT* promoter-mutated MLS. We report the prevalence of *TERT* promoter mutations (overall incidence near to 9.5%) to vary widely among different bone and STS subtypes (ranging from 0–44%), with the highest mutation frequency found within MLS subgroup (44% harboring a C228T mutation at the promoter region of the *TERT* gene). The two existing mutations C228T and C250T were found to be mutually exclusive in every case. Our results add value to previous studies on smaller cohorts or limited number of sarcoma subtypes, as those same hotspot mutations have also previously been characterized as recurrent in MLS but rather uncommon in other subtypes (Table 1) [13,37,38,39]. However, the mutational frequency in the MLS subgroup appears to fluctuate substantially among studies (22–23% vs. 40% vs. 74–79%) apparently due to differences in the size of the cohorts (Table 1) [13,37,38,39]. Of note, the C228T is the predominant mutation found in our sarcoma cohort similar to the results of previous studies [13,37,38,39]. Yet, we describe the first case of a C250T *TERT* promoter mutation in a chondrosarcoma, after Killela et al. [13] who reported the other known published case.

*TERT* promoter mutations occur mainly in tumors that are derived from tissues with low rates of self-renewal [13,14]. In line with this observation, we found significant more *TERT* promoter mutations in G1 tumors than in G2–3 tumors. The high frequency of *TERT* promoter mutations in just two nucleotide positions strongly suggests a possible role as drivers, i.e., primary or secondary events in tumor pathogenesis [12]. Chiba et al. have indeed shown that *TERT* promoter mutations are sufficient to overcome the proliferative barrier imposed by critical telomere shortening without additional tumor-selected mutations [40]. In addition, some studies suggest *TERT* promoter mutations are among the earliest genetic events in bladder cancer [41], thyroid carcinoma [42], cutaneous melanoma [14,43,44], basal cell and squamous cell carcinoma [45] and oligodendroglioma [46]. In contrast, other studies implicate that the occurrence of *TERT* mutations is more likely a secondary genetic event following the activation of an oncogenic signaling pathway, such as *MAPK* signaling in melanoma [14] or Wnt signaling in hepatocellular carcinoma [47].

Based on these observations, we aimed to analyze the impact of the *TERT* promoter mutations on telomere lengths. Therefore, we used MLS as an ideal model to study telomere lenght in sarcoma as this sarcoma subtype is not only characterized by a typical initial oncogenic driver translocation, i.e., the t(12;16)(q13; p11) [26,48], but also by a substantially higher incidence of *TERT* promoter mutations compared to other sarcomas subtypes [13,37,38,39]. Our telomere analysis on MLS histopathological slides revealed significantly less shortened telomeres in MLS with *TERT* promoter mutations compared to wildtype tumors after age matching. Of note, the telomere length of wildtype tumor was close to the range where telomeres become critically short (known to be near 3–5 kb [49,50,51,52]) as e.g., similar to the telomere length of patients with dyskeratosis congenita, a disease characterized by mutations within the telomerase complex [53]. In line with our telomere data, Kabjorn et al. found that MLS typically display a major population of senescent cells [54]. It is tempting to speculate that in some of the cases described critically short telomeres might contribute to cell senescene due to unresolved telomere crisis.

One explanation for our observations might be the upregulation of telomerase activity by frequently occurring *TERT* promoter mutations leading to telomere elongation/maintenance in *TERT* promoter-mutated tumors. Due to poor quality of the RNA extracted from older paraffin embedded tumor samples in our patient cohort, analysis of *TERT* expression and/or telomerase activity is extremely limited to further support our hypothesis. Other mechanisms for telomere elongation as e.g., ALT do not play an important role in our patient cohort, in line with the observed low frequency (approx. 5–13%) estimated by other studies [33,34]. No data is available with regard to the possibility of increased expression of C/EBP—due to FUS-DDIT3—increasing *TERT* expression in tumor cells. However, the high incidence of *TERT* promoter mutations in MLS implies that C/EBP has no major role in telomerase regulation and telomere maintenance.

Alternatively, *TERT* promoter mutations preferentially occur in tumor cells with longer telomeres or occur before the typical initial oncogenic driver translocation. The latter seems less likely due to substantially higher incidence of oncogenic driver as opposed to (presumably secondary) *TERT* promoter mutations. To clarify this issue, telomere length and *TERT* promoter status of the tissue from where MLS originates should be analyzed. However, the cell-of-origin in MLS sarcomas, most likely transformed mesenchymal stem cells and/or their immediate lineage progenitors [55,56], remains unknown, thus limiting definitive conclusions.

Based on our experimental data, we propose that the acquisition of *TERT* promoter mutations is a secondary event in disease progression of MLS required to protect MLS cells from growth arrest due to replication-induced critically short telomeres (see proposed model in Figure S2)

Although is it evident that *TERT* promoter mutation status and telomere regulation is variable and complex among different cancers types, particularly due to the variety related to tumor cell origin, tumor subtype [29,30,31,32,33,34,35,36,37,38,39,57], our proposed model is further substantiated by others studies. In follicular and atypical thyroid adenoma [42] as well as gliomas, *TERT* promoter mutations lead to significantly increased telomerase activity [12,13,58] explaining longer telomeres observed in our cohort of mutated MLS. Similarly, in bladder cancer it has been shown that *TERT* promoter mutations promote a mechanism for high-level telomerase reactivation associated with stable telomere length [59].

*TERT* promoter mutations alone have been linked with worse prognosis in various tumor entities such as melanoma [60,61], glioblastoma multiforme [13], medulloblastoma [62], urogenital cancer [63,64], laryngeal tumors [65], and with larger tumors and lymph node metastasis in the case of conventional papillary thyroid carcinomas [12,66,67,68]. Unfortunately, the low number of MLS cases in our study did not allow correlations with clinical outcome. Based on the role of *TERT* promoter mutations in other tumor entities and the perception that its occurrence is a secondary event in MLS, it is tempting to see clinical similarities between MLS and e.g., gliomas [69]. However, further studies are needed to unravel the role of *TERT* promoter mutations in MLS.

In summary, we observed that in a subset of MLS patients, *TERT* promoter mutations likely occur as a secondary event in a setting of critically short telomere length, most likely reflecting a compensatory mechanism that allows telomeres to elongate/maintain. Further studies are needed to further analyze the impact of *TERT* promoter mutations and telomere length on clinical outcome.

## 4. Material and Methods

### 4.1. Sarcoma Samples

Formalin-fixed paraffin-embedded (FFPE) slices of 116 patients diagnosed between 2007 and 2014 with a large variety of histological subtypes of bone and STS were analyzed. Samples were obtained from diagnostic or surgical resections from the archive of the Institute of Pathology of the University Hospital RWTH Aachen. Informed consent for retention and analysis of the tissue for research purposes was obtained from all patients (local ethical review board of the Medical Faculty of the RWTH Aachen University, EK-206/09). In this study, the following sarcoma subtypes were included: undifferentiated pleomorphic sarcoma (*n* = 9); dedifferentiated liposarcoma (*n* = 12); well differentiated liposarcoma (*n* = 5); pleomorphic sarcoma (*n* = 4); liposarcoma (*n* = 7); MLS (*n* = 9); sarcoma not otherwise specified (NOS, *n* = 11); synovial sarcoma (*n* = 9); chrondroma (*n* = 1); chondrosarcoma (*n* = 9); osteosarcoma (*n* = 3); rhabdomyosarcoma (*n* = 8); leiomyosarcoma (*n* = 9); myofibroblastic sarcoma (*n* = 3); myxofibrosarcoma (*n* = 1); malignat peripheral nerve sheath tumor (*n* = 8); solitary fibrous tumor (*n* = 1); clear cell sarcoma (*n* = 1); Ewing sarcoma (*n* = 1); Cytosarcoma phyllodes (*n* = 1); epitheloid haemangiosarcoma (*n* = 1); and angiosarcoma (*n* = 3). Tumors were staged according to the TNM classification of malignant tumors (TNM) staging system of the Union for International Cancer Control (UICC). Detailed clinical patient characteristics are summarized in Table 2. For *TERT* promoter-mutated patients additional parameters are shown in Table 3.

### 4.2. Polymerase Chain Reaction (PCR) Amplification and Sanger Sequencing

One single 10 µm slice was prepared from each FFPE tumor block and used for extraction of genomic DNA. Extraction was done using the GeneRead DNA FFPE kit (Qiagen, Germany), according to the manufacturer’s instructions. The extracted DNA was quantified by spectrophotometry using Nanodrop spectrophotometer (Thermo Fischer, Schwerte, Germany). The protocol for polymerase chain reaction (PCR) amplification followed previously published protocols [10]. Briefly, 150 ng of DNA template in a total volume of 50 µL were used, 2.5 U/reaction Taq Biotherm DNA polymerase (Genecraft, Köln, Germany) were used and cycling conditions included initial denaturation at 94 °C for 120 s, followed by 35 cycles with denaturation at 94 °C for 60 s, annealing at 60 °C for 30 s and extension at 72 °C for 30 s. PCR primers amplifying a fragment of the *hTERT* promoter region containing the sites of the c.-124 C>T (C228T) and c.-146 C>T (C250T) mutations were used to screen for mutations. The primers hTERT-286-R: 5′-CTCCCAGTGGATTCGCGGGC-3′ and hTERT-27-F: 5′-CCCACGTGCGCAGCAGGAC-3′, yielding a 260 bp product, were used for all samples. When amplification of the larger fragment failed, the alternative primers hTERT-200-R: 5′-CACCCGTCCTGCCCCTTCACCTT-3′ and hTERT-26-F: 5′-GGCTTCCCACGTGCGCAGCAGGA-3, yielding a 192 bp product, were used. A run 1.6% agarose gel was used to confirm the presence of amplified products. PCR products were clean with the Clean & Concentrator Kit-5 (Zymo Research, Irvine, KY, USA) before being sent to Eurofins Genomics (Ebersberg, Germany) for Sanger sequencing analysis to confirm the presence/absence of mutations. Analysis of chromatograms and sequences was performed with GENtle 2.0 software (open source).

### 4.3. Telomere Length Analysis by Quantitative In Situ Hybridization (Q-FISH)

For quantitative in situ hybridization (Q-FISH), 5-µm archive FFPE slices were prepared and stained as previously published [49,70,71,72,73]. Deparaffinized and dehydrated sections were incubated with a Cy3-(C3TA2)-PNA (Panagene, Daejeon, Korea) probe for 2 h before being washed and nuclei counterstained using 0.1 µg/mL DAPI (Sigma, München, Germany). Stained slides were stored at 4 °C and laser confocal microscopy images captured within 72 h after sample processing. Telomere signals were acquired with a Zeiss LSM710 (Jena, Germany) confocal laser-scanning microscope. Images were captured at optical magnification of 63×, with additional 1.2× digital zoom under multi-tracking mode with 1 µm steps (maximum intensity projections of 5 steps were prepared). Five tumorigenic areas (>100 cells total) were captured for each slice. Patients with or without *TERT* promoter mutation were assessed in parallel. Telomere length was quantified using Definiens Developer Software (Definiens 2.3, München, Germany). Nuclei and telomeres were detected based on the respective DAPI and Cy3 intensities. Telomere length was calculated in kb using internal controls as described previously [49,70,71,72,73].

### 4.4. Alternative Lengthening of Telomeres (ALT) Assessment

The presence of ALT phenotype was assessed by the identification of very large and bright intranucelar foci of telomere FISH signals after Q-FISH staining. Criteria for ALT positivity was ≥1% tumor cells displaying ultra-bright intranuclear foci of telomere FISH signals with foci intesity being 10-fold higher than mean intesity of all telomeric signals in non-tumor stroma cells wihtin each sample [74]. A glioblastoma sample was included as positive control for the presence of ALT.

### 4.5. Statistical Analysis

GraphPad Prism 5.0 (GraphPad, La Jolla, CA, USA) was used for statistical analysis. Differences in telomere length between the groups were evaluated by unpaired *t*-testing. Chi-square test was used to compare patient’s characteristics between WT and *TERT* promoter-mutated groups. *p* < 0.05 was statistically significant.

## Figures and Tables

**Table 1:** Overview of the frequency of *TERT* promoter mutations in soft tissue and bone sarcomas reported in the literature: Number of mutated cases/total cases (mutation rate, in percentage).

	Present Study (*n* = 116) 22 Subtypes	Saito 2016 (*n* = 180) 19 Subtypes	Campanella 2016 (*n* = 68) 15 Subtypes	Koelsche 2014 (*n* = 341) 16 Subtypes	Killela 2013 (*n* = 95) 6 Subtypes
**Soft tissue sarcomas**					
WDLS	0/5 (0%)	0/18 (0%)	0/1 (0%)	NA	NA
Myxoid liposarcoma	4/9 (44%)	3/13 (23%)	2/9 (22.2%)	29/39 (74%)	19/24 (79.1%)
Pleomorphic liposarcoma	1/4 (25%)	NA	1/1 (100%)	0/15 (0%)	NA
Dedifferentiated liposarcoma	0/12 (0%)	NA	1/1 (100%)	0/61 (0%)	NA
Liposarcoma	0/7 (0%)	NA	NA	NA	NA
Leiomyosarcoma	0/9 (0%)	0/24 (0%)	0/13 (0%)	0/27 (0%)	NA
Cytosarcoma Phyllodes	0/1 (0%)	NA	NA	NA	NA
Angiosarcoma	0/3 (0%)	NA	NA	0/9 (0%)	NA
Rabdomyosarcoma	0/8 (0%)	0/5 (0%)	NA	NA	NA
Haemangiosarcoma	0/1 (0%)	NA	NA	NA	NA
MPNST	0/8 (0%)	0/1 (0%)	0/7 (0%)	2/35 (6%)	NA
UPS (previous MFH)	2/9 (22.2%)	1/22 (4.5%)	0/12 (0%)	0/40 (0%)	NA
Fibrosarcoma	NA	NA	0/1 (0%)	NA	1/3 (33.3%)
Myofibroblastic sarcoma	0/3 (0%)	NA	0/3 (0%)	NA	NA
Myxofibrosarcoma	0/1 (0%)	0/6 (0%)	0/5 (0%)	0/17 (0%)	1/10 (10%)
NOS	0/11 (0%)	NA	NA	NA	NA
NOS, poorly differentiated	1/8 (12.5%)	NA	NA	NA	NA
Clear cell sarcoma	0/1 (0%)	0/1 (0%)	0/1 (0%)	0/5 (0%)	NA
Synovial sarcoma	0/9 (0%)	0/7 (0%)	0/9 (0%)	1/25 (4%)	NA
Ewing sarcoma	0/1 (0%)	0/6 (0%)	NA	NA	NA
SFT	1/1 (100%)	5/40 (12.5%)	0/1 (0%)	4/31 (13%)	2/10 (20%)
**Bone sarcomas**					
Chondrosarcoma	2/8 (25%)	0/10 (0%)	NA	0/8 (0%)	1/2 (50%)
Osteosarcoma	0/3 (0%)	0/14 (0%)	NA	NA	1/23 (4.3%)
Overall	11/116 (9.5%)	9/167 (5.4%)	4/68 (5.9%)	36/341 (10.5%)	25/72 (34.7%)

NA—not available; WDS—well-differentiated liposarcoma; UPS—undifferentiated pleomorphic sarcoma; MPNST—malignant peripheral nerve sheath tumor; SFT—solitary fibrous tumor; MFH—malignant fibrous histiocytoma; NOS—not otherwise specified; UPS—undifferentiated pleomorphic sarcoma.

**Table 2:** Clinical characteristics of the selected cohort of soft tissue and bone sarcoma patients.

Characteristics (*n* = 116)	WT (*n*)	TERTp (*n*)
Age (in years)		
≤51	39	4
>51	66	7
Gender		
Male	57	7
Female	48	4
Race		
Caucasian	99	11
Non Caucasian	6	0
Tumor location		
Abdominal	16	0
Arm/forearm	7	0
Cervical	5	0
Femoral	11	3
Head	3	0
Hip	5	1
Jaw	2	1
Knee	1	1
Leg	4	0
Lung	8	1
Retroperitoneal	18	0
Spine	2	1
Testis	4	0
Thigh	4	1
Others	15	2
Grade		
G1/well differentiated	16	5
G2-G3	86	6
NA	3	0
Disease progression		
No	53	5
Yes	45	3
NA	7	3
Metastasis		
Absent	55	6
Present	42	2
NA	8	3

N—number of cases. Tumor location “Others” included bladder, ear, gluteus, groin, liver, maxilla, nose, paravertebral, parotis, pleural, prostate, renal, rip/sternum, shoulder, skin, Vena cava. NA—not available. Disease progression during follow-up. Metastasis during follow-up.

**Table 3:** Details of *TERT* promoter (*TERTp*) mutated-patients.

Diagnosis	Age/Gender	Location	TERTp Mutation	Tumor Grade	Metastasis	Progression
MLS	61/F	thigh	C228T	G1	NA	NA
MLS	71/M	femoral	C228T	G1	yes	yes
MLS	23/M	lung	C228T	G1	NA	NA
MLS	43/F	spine	C228T	G2	yes	yes
PS	58/F	femoral	C228T	G2	no	no
MFH	68/M	skin	C228T	G2	no	no
MFH	78/M	hip	C228T	G3	yes	no
CS	37/M	femoral	C228T	G2	no	no
CS	46/F	jaw	C250T	G1	no	no
SFT	64/F	NA	C228T	well diff.	yes	yes
NOS	68/M	ear	C228T	G2	no	no

Age given in years. MLS—myxoid liposarcoma; PS—pleomorphic sarcoma; MFH—malignant fibrous histiocytoma; CS—chondrosarcoma; SFT—solitary fibrous tumor; NOS—not otherwise specified; NA—not available; well diff.—well-differentiated tumor. Metastasis and disease progression during follow-up.

## Supplementary Materials

The following are available online at http://www.mdpi.com/1422-0067/19/2/608/s1.
