# Supplementary material for: Presence of TERT Promoter Mutations is a Secondary Event and Associates with Elongated Telomere Length in Myxoid Liposarcomas
Source: Int J Mol Sci. 2018 Feb 18;19(2):608. doi: 10.3390/ijms19020608 (PMC5855830; doi:10.3390/ijms19020608)
Supplement: Supplementary file 1 [file ijms-19-00608-s001.pdf]

International Journal of *Molecular Sciences*

Supplementary Materials

Presence of *TERT* Promoter Mutations is a Secondary Event and Associates with Elongated Telomere Length in Myxoid Liposarcomas

Monica S. Ventura Ferreira [1], Martina Crysandt [1], Till Braunschweig [2], Edgar Jost [1], Barbara Voss [1], Anne-Sophie Bouillon [1], Ruth Knuechel [2], Tim H. Brümmendorf [1] and Fabian Beier [1,*]

Received: 12 January 2018; Accepted: 10 February 2018; Published: 18 February 2018

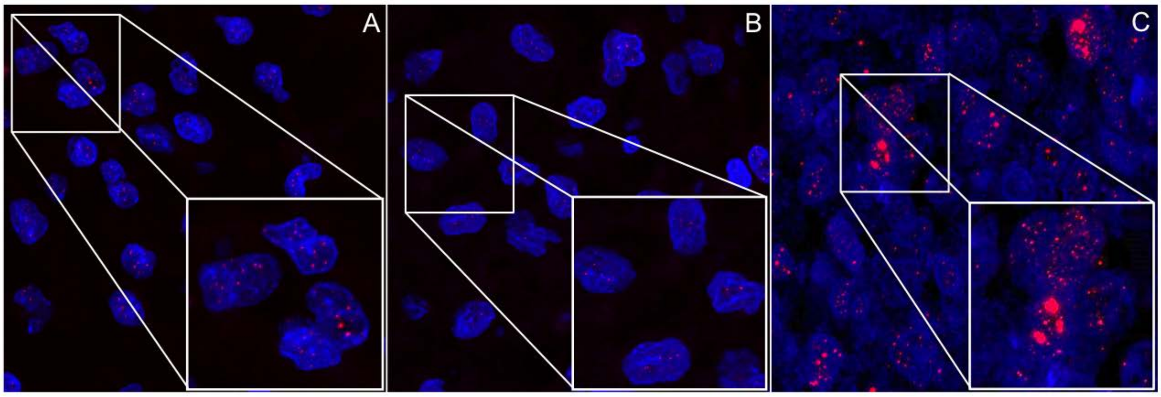

Figure S1. Representative telomere Q-FISH images of (A) C228T TERT promoter mutated myxoid liposarcoma, (B) TERT promoter wildtype myxoid liposarcoma, (C) ALTpositive glioblastoma control. Magnifications 630x.

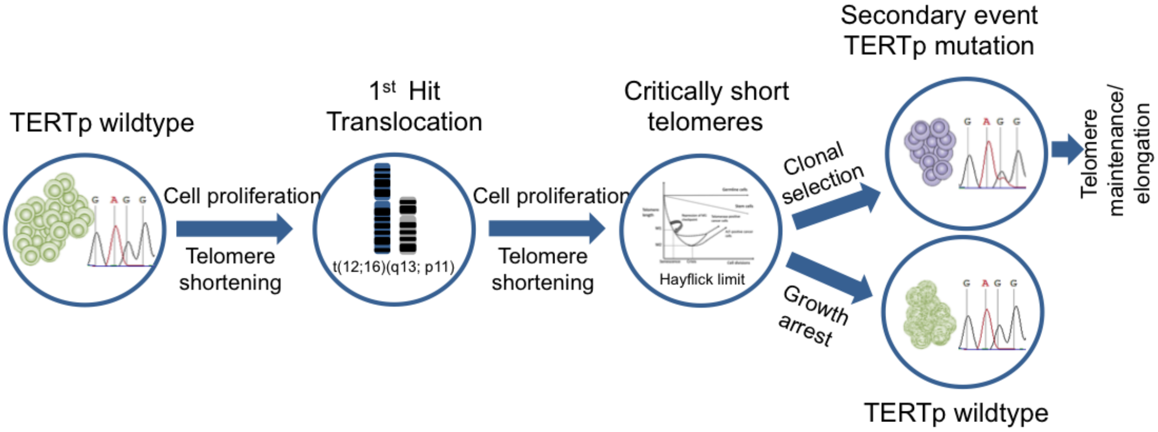

Figure S2. Schematic for the hypothesized subsequent events after 1st hit acquisition (genetic translocation) in myxoid liposarcoma (MLS). Wildtype tumors go into telomere crisis as their telomere lengths reach the "Hayflick limit". Critically short telomeres lead to growth arrest. In MLS tumors, there is activation of a telomere maintenance mechanism that allows for cell selection by clonal advantage. A secondary event, i.e., the 13 acquisition of a TERT promoter mutation occurs initiating a compensatory telomere response mechanism that maintain/elongate telomeres.
